# Bio-ethanol production through simultaneous saccharification and co-fermentation (SSCF) of a low-moisture anhydrous ammonia (LMAA)-pretreated napiegrass (*Pennisetum purpureum* Schumach)

**DOI:** 10.1186/2193-1801-3-333

**Published:** 2014-07-01

**Authors:** Masahide Yasuda, Hayato Nagai, Keisuke Takeo, Yasuyuki Ishii, Kazuyoshi Ohta

**Affiliations:** Department of Applied Chemistry, Faculty of Engineering, University of Miyazaki, Gakuen-Kibanadai Nishi, Miyazaki, 889-2192 Japan; Department of Animal and Grassland Sciences, Faculty of Agriculture, University of Miyazaki, Gakuen-Kibanadai Nishi, Miyazaki, 889-2192 Japan; Department of Biochemistry and Applied Biosciences, Faculty of Agriculture, University of Miyazaki, 1-1 Gakuen-Kibanadai Nishi, Miyazaki, 889-2192 Japan

**Keywords:** Bio-ethanol, Simultaneous saccharification and co-fermentation (SSCF), Napiergrass, Cellulase, Xylanase, *Escherichia coli* KO11, LMAA-pretreatment

## Abstract

Efficient bio-ethanol production from napiegrass (*Pennisetum purpureum* Schumach) was investigated. A low-moisture anhydrous ammonia (LMAA)-pretreated napiegrass was subjected to simultaneous saccharification and co-fermentation (SSCF), which was performed at 36°C using *Escherichia coli* KO11, *Saccharomyces cerevisiae*, cellulase, and xylanase. It was found that use of xylanase as well as the LMAA-pretreatment was effective for the SSCF. After the SSCF for 96 h, the ethanol yield reached 74% of the theoretical yield based on the glucan (397 mg g^-1^) and xylan (214 mg g^-1^) occurring in dry powdered LMAA-pretreated napiergrass.

## Introduction

Bioethanol from lignocellulosic biomass has been recognized as one of promising sustainable energy source alternative to petroleum-based fuels, since the lignocelluloses are not directly in competition with food sources (Galbe and Zacchi 2007; Taherzadeh and Karimi 2007). We are interested in ethanol production from herbaceous lignocellulosic napiergrass (*Pennisetum purpureum* Schumach) because of its low lignin content and high harvest amount per year and per area (Yasuda et al. 2012, 2013). In lignocellulosic ethanol production, pentose fermentation is an unavoidable process because of its high xylan content. In pentose fermentation process, however, ethanol concentration is usually too low (<10 g L^-1^) to distillate with low energy cost. Therefore, pentose fermentation has been performed as co-fermentation of hexose and pentose using a variety of recombinant strains such as *Escherichia coli* KO11 (Ohta et al. 1990, 1991; Underwood et al. 2002; Brandon et al. 2011), *Saccharomyces cerevisiae* 424A(LNH-ST) (Jin et al. 2012a, [b]), *S. cerevisiae* TMB3400 (Ohgren et al. 2006; Matsushika et al. 2009), and *Zymomonas mobilis* AX101 (Su et al. 2012). Simultaneous saccharification and co-fermentation (SSCF) is generally superior to the separate saccharification and co-fermentation since SSCF can achieve saccharification, hexose fermentation, and pentose fermentation in one-pot reaction. Here, we applied SSCF to ethanol production from napiergrass, which was treated by low-moisture anhydrous ammonia (LMAA) pretreatment.

## Materials and methods

### Napiergrass as lignocellulosic materials

As raw material, a dwarf type of napiergrass was cultivated in the Kibana Agricultural Science Station, at the University of Miyazaki. Leaf blades of the napiergrass were separated from the stem and then cut by a cutter and dried at 70°C for 72 h. The dried matter was ground until 70% of the particles were in a range of 32–150 μm in length.

### Chemical components of napiergrass

The powdered napiergrass (30 g) was treated with a 1% aqueous solution of NaOH (400 mL) at 95°C for 1 h. The holocellulose was isolated as a pale yellow precipitate by centrifugation and filtration of the treated mixture. The supernatant solution was neutralized to pH 5.0 by a dilute HCl solution. The resulting dark brown precipitate identified as lignin was collected via centrifugation at 10,000 rpm for 10 min. Sugars in holocellolose were determined according to the methods published by the National Renewable Energy Laboratory as follows (Sluiter et al. 2010). Sulfuric acid (72 wt%, 3.0 mL) was added slowly to holocelulose (300 mg) and kept at 30°C for 1 h. The resulting solution was diluted by water (84 mL) until the concentration of sulfuric acid was 4 wt%. Acid hydrolysis was performed by autoclaving at 121°C for 1 h in an autoclave. After the neutralization by CaCO_3_, the solution was subjected to the centrifugation to give the supernatant solution (ca. 87 mL), which was concentrated to 30 mL by evaporation. The solution was analyzed by HPLC. The peaks of glucose and xylose appeared whereas the peaks of galactose and arabinose were very weak because of their low contents. The amounts of glucan and xylan were determined from the amounts of glucose and xylose determined by HPLC. It was confirmed that the sum amounts of glucan and xylan were equaled to the amounts of hollocellolose. The ash component in lignocellulose was obtained by the burning of the lignocellulose (2.0 g) in an electric furnace (KBF784N1, Koyo, Nara, Japan) for 2 h at 850°C.

### Low-moisture anhydrous ammonia (LMAA) pretreatment

Water (100 g) was added dropwise to dry powdered napiergrass (100 g, volume 320 mL) in the flask (1 L). The resulting moist powdered napiergrass in the flask was evacuated with a pump under 20 mm Hg and then gaseous NH_3_ was introduced into the flask. This operation was performed three times until the atmosphere inside the flask was entirely replaced with NH_3_. The amount of NH_3_ presented in the flask was 1.1 g. The LMAA pretreatment was performed by modifying the Kim method where LMAA pretreatment was performed at 80°C for 86 h (Yoo et al. 2011). In our LMAA-pretreatment, the moist powdered napiergrass was kept under NH_3_ gas atmosphere at room temperature for four weeks (28 days). After the treatment, the NH_3_ was removed with an evaporator. The treated napiergrass was washed with water (2 L) three times to separate the brownish aqueous solution of the lignin. After pH was checked to be neutral, the pretreated napiergrass was dried at 60°C to weigh out the precise amount of napiergrass in the following biological treatment.

### Hydrolytic enzyme for saccharification

A cellulase from *Acremonium cellulolyticus* (Acremozyme KM, Kyowa Kasei, Osaka, Japan) was selected by comparing its activity with other cellulases such as Meycellase (Meiji Seika), a cellulase from *Trichoderma viride* (Wako Chemicals, Osaka, Japan) and a cellulase from *Aspergillus niger* (Fluka Japan, Tokyo) (Yasuda et al. 2011). The cellulase activity of Acremozyme KM was determined to be 1,320 units mg^–1^ by the method of breaking down filter paper (Kitamikado and Toyama 1962). A xylanases from *Trichoderma longibrachiatum (reesei)* (Sumizyme X, Shin Nihon Chemicals, Anjyo, Japan, 5,000 u g^-1^) was selected from commercially available hemicellulase.

Saccharification was performed for the powdered napiergrass (10.0 g) using both cellulase and xylanase, whose total amount was 1.0 g, at 45°C in an acetate buffer (60 mL, pH 5.0).

### Preparation of the inoculum culture of *Escherichia coli*KO11 and *Saccharomyces cerevisiae*

*E. coli* KO11 was grown in the LB medium (200 mL) consisting of tryptone (2.0 g L^–1^, Difco), yeast extract (1.0 g L^–1^), and NaCl (2.0 g L^–1^) under shaking at 150 rpm at 37°C for 24 h. The KO11 cell suspension contained a dry weight of 0.52 mg mL^–1^ of *E. coli* KO11. *Saccharomyces cerevisiae* NBRC 2044 was grown at 30°C for 24 h in a basal medium (initial pH 5.5) consisting of glucose (20.0 g L^–1^), polypeptone (1.0 g L^–1^), yeast extract (1.0 g L^–1^), KH_2_PO_4_ (1.0 g L^–1^), and MgSO_4_ (3.0 g L^–1^). After incubating for 24 h, the cell suspension of *S. cerevisiae*, whose grown culture of *S. cerevisiae* showed a cell density of 7.7 × 10^7^ cells mL^–1^, was obtained (Yasuda et al. 2012).

### Simultaneous saccharification and co-fermentation (SSCF)

Typical procedure of SSCF is as follows. The LMAA-pretreated napiergrass (3.0 g) was suspended in the acetate buffer (14.0 mL, pH 5.0) and then autoclaved at 121°C for 20 min. After cooling to room temperature under UV-irradiation, the cell suspension (0.36 mL) of *S. cerevisiae,* a portion (21 mL) of the inoculum culture *E. coli* KO11, and the cellulase (150 mg) and xylanase (150 mg) in an acetate buffer solution (5.0 mL, pH 5.0) were added to the suspension of the napiergrass. After pH was adjusted to 6.0, air was purged with N_2_. In the SSCF without *S. cerevisiae*, replacement by N_2_ gas was not performed. The SSCF was initiated by stirring the solution vigorously with a magnetic stirrer at 36°C which was an optimal fermentation temperature of *E. coli* KO11. The evolved CO_2_ was collected over water by a measuring cylinder, and the reaction was monitored by the volume of CO_2_. The SSCF reaction was continued for 96 h until CO_2_ evolution ceased.

### Analytical methods

Saccharides were analyzed on a high-performance liquid chromatography system (LC-20AD, Shimadzu, Kyoto, Japan) equipped with RI detector (RID-10A) using an anion exchange column (NH2P-50 4E; Shodex Asahipak, 250 mm in length and 4.6 mm in ID, Yokohama, Japan). Acetonitrile-water (8:2 v/v) was flowed at 1.0 mL min^-1^ as mobile phase. Ethanol was analyzed by gas-liquid chromatography using 2-propanol as an internal standard on a Shimadzu gas chromatograph GC-8A equipped with a glass column of 5% Thermon 1000 on Sunpak-A (Shimadzu).

## Results and discussion

### Ethanol production from lignocelluloses

In general, the cellulosic bio-ethanol production involves three steps such as saccharification of cellulosic components (SA), hexose fermentation (HF), and pentose fermentation (PF). These processes are combined each other to simplify the procedure and enhance the ethanol yield. Typical combinations are as follows: SSF is simultaneous process of SA and HF but does not take place PF. CF is co-fermentation of hexose and pentose. SSCF is simultaneous process of SA, HF, and PF. For efficient cellulosic bio-ethanol production, moreover, pretreatment to remove the lignin and/or promote an enzymatic digestibility of the cellulosic components are usually required.

### Napiergrass as raw material

Napiergrass belongs to herbaceous tropical species, native to the east Africa and has high dry matter productivity with moderate forage quality in southern Kyushu (Ishii et al. 2005a, 2013). Napiergrass has wide variation of phenotypes, reflected by plant breeding due to the crossing of dwarf genotype and relative species such as pearl millet (*Pennisetum americanum*) (Ishii et al. 2005a; Hanna and Sollenburger 2007). A dwarf variety of late-heading type of napiergrass (dwarf napiergrass) originated from Florida, USA, via Thailand (Mukhtar et al. 2003) was assessed to be suitable for both grazing (Ishii et al. 2005b) and cut-and-carry systems among several sites of southern Kyushu, Japan (Utamy et al. 2011). Dwarf napiergrass meets the requirement of lignocellulose for the biofuel production because it has low lignin-content and a relatively high herbage mass per year and per area (Rengsirikul et al. 2011; Rengsirikul et al. 2013; Khairani et al. 2013). Therefore, we have continued to use this dwarf type of napiergrass for the bio-ethanol (Yasuda et al. 2011) and bio-hydrogen production (Shiragami et al. 2012).

As has been reported previously (Yasuda et al. 2013), the LMAA-pretreatment was useful for the simultaneous saccharification and fermentation (SSF) of napiergrass. In the present case, therefore, the powdered napiergrass was subjected to the LMAA-pretreatment. The LMAA-pretreated napiergrass contained 39.7 wt% of glucan, 21.4 wt% of xylan, 7.1 wt% of lignin, and 7.1 wt% of ash, while the components of the non-treated napiergrass were determined to be 31.3 (glucan), 16.9 (xylan), 12.6 (lignin), and 13.9 wt% (ash).

### Optimization of enzymatic saccharification

**Figure 1:**
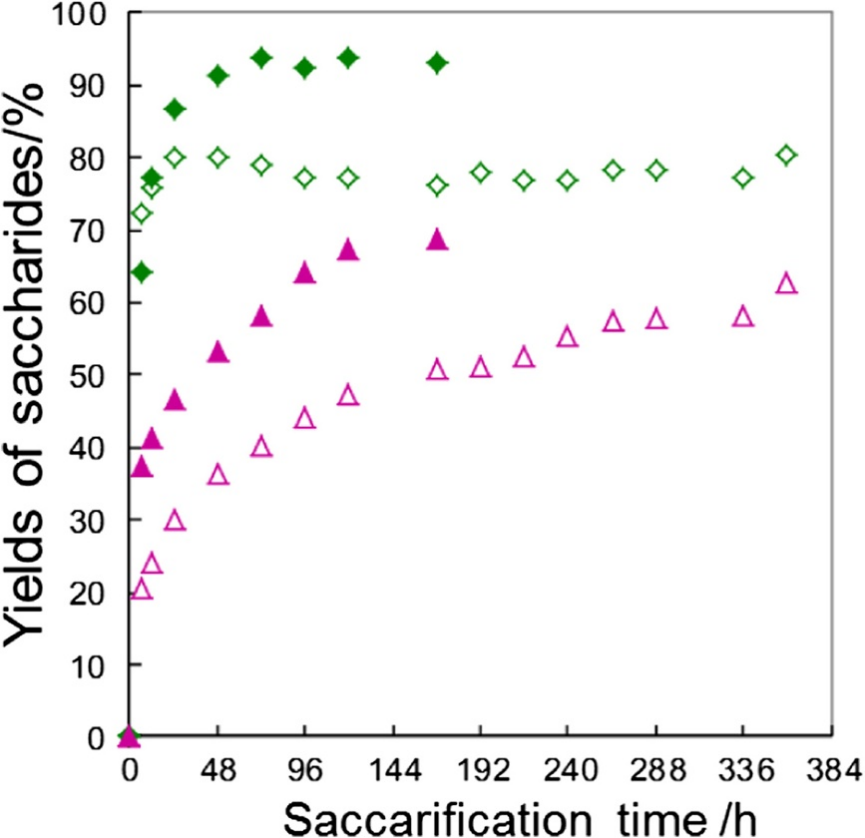
**Time-conversion plots of the amounts of glucose and xylose in saccharification of LMAA-pretreated napiergrass (10.0 g) using cellulase and/or xylanase.** Glucose (◆) and xylose (▲) from the saccharification using cellulase (0.50 g) and xylanase (0.50 g). Glucose (◇) and xylose (△) from the saccharification using only cellulase (1.0 g).

The effect of LMAA-pretreatment was checked by the enzymatic saccharification. Saccharification of non-treated and LMAA-pretreated napiergrass (10.0 g) was performed using cellulase (Acremozyme KM, 1.00 g) at 45°C in an acetate buffer (60 mL). Results are shown in Table 1. Saccharification of no-treated napiergrass produced the total saccharides in 54% yield (Table 1 run 1). In the case of napiergrass which was subjected LMAA pretreatments for four weeks, the yield of saccharides was 67% which was the higher than the cases of LMAA for one and two weeks (runs 2–4). Figure 1 shows the time-conversion of glucose and xylose obtained from the saccharification of the LMAA-pretreated napiergrass. Although the glucose increased rapidly to reach the maximum amounts until 48 h, xylose increased gradually even at saccharification for 360 h. Thus the saccharification of xylan was very slow.

**Table 1:** **Saccharification of LMAA-pretreated napiergrass** ^**a)**^

Run	PT ^b)^	***F***_X_^c)^	Product/g (yield/%) ^d)^		
			Glucose	Xylose	Total
1	NO	0.0	2.20 (63)	0.68 (36)	2.88 (54)
2	LMAA (1)	0.0	2.89 (66)	0.91 (38)	3.80 (63)
3	LMAA (2)	0.0	3.07 (70)	1.16 (49)	4.23 (63)
4	LMAA (4)	0.0	3.36 (76)	1.20 (51)	4.57 (67)
5	LMAA (4)	0.3	3.23 (73)	1.13 (48)	4.36 (64)
6	LMAA (4)	0.4	3.56 (81)	1.49 (63)	5.05 (74)
7	LMAA (4)	0.5	4.14 (94)	1.60 (68)	5.74 (85)
8	LMAA (4)	0.6	4.25 (96)	1.48 (62)	5.73 (86)
9	LMAA (4)	0.7	3.92 (90)	1.51 (64)	5.43 (81)
10	LMAA (4)	0.8	3.72 (84)	1.47 (62)	5.20 (78)
11	LMAA (4)	0.9	3.63 (82)	1.30 (55)	4.93 (73)
12	LMAA (4)	1.0	3.05 (69)	0.95 (40)	4.00 (59)

^a)^Saccharification was performed for napiergrass (10.0 g) using the hydrolytic enzyme (1.0 g) in an acetate buffer (60 mL) at 45°C for 168 h.

^b)^Pretreatment (PT). NO: non-treatment. LMAA: a low-moisture anhydrous ammonia pretreatment. The value in parenthesis was the period in week for LMAA- pretreatment.

^c)^ *F*_X_ value was the fraction of xylanase in the mixture (1.0 g) of cellulase and xylanase.

^d)^The amounts of saccharides obtained from the saccharification of 10 g of the pretreated napiergrass.

**Figure 2:**
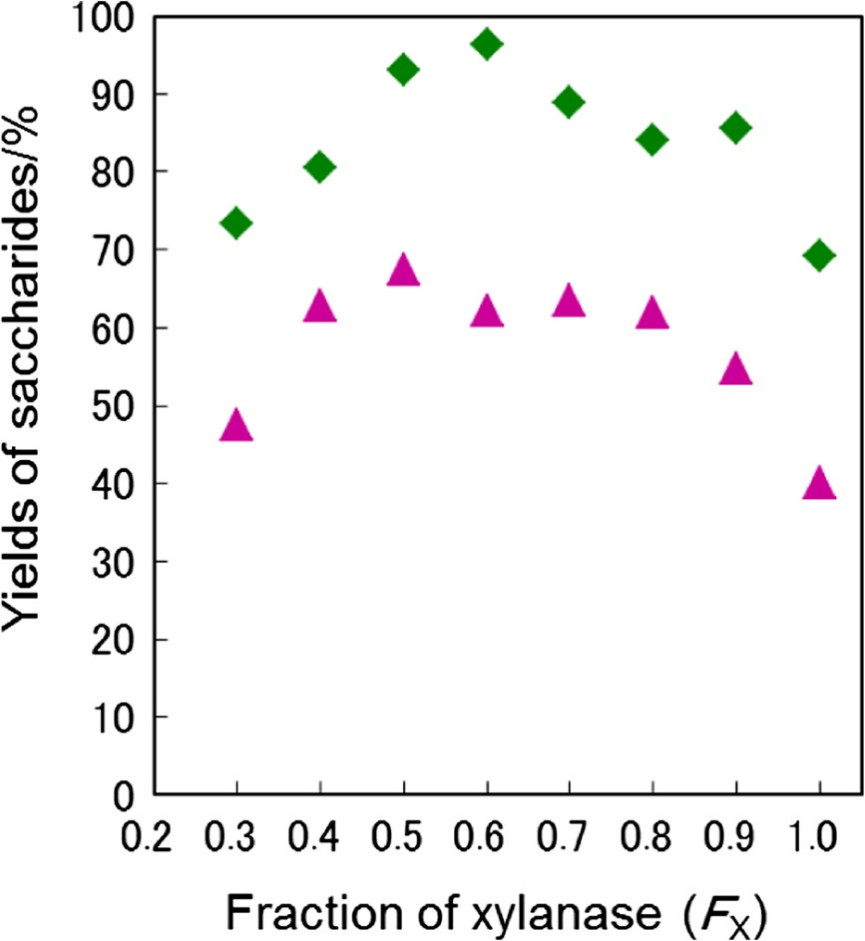
**The effects of the fraction of xylanase (** ***F***_**X**_ **) on the yields of glucose (**◆**) and xylose (**▲**).** The saccharification of LMAA-pretreated napiergrass (10.0 g) was performed for 168 h using a mixed enzyme of cellulase and xylanase.

In order to shorten the saccharification time, xylanase (Sumizyme X) was used in addition to cellulase. Xylanase was used in *F*_X_ × 1.0 g where *F*_X_ was a fraction of xylanase in the mixed enzymes. Figure 2 shows the effect of *F*_X_ on the saccharide yields in the saccharification of LMAA-pretreated napiergrass for 168 h. The optimal *F*_X_ value was determined to be 0.50 since the yield of xylose was maximum. Under optimized conditions where the saccharification was performed for the LMAA-pretreated napiergrass (10.0 g) by a mixture of enzymes, cellulase (0.50 g) and xylanase (0.50 g), the yields of glucose and xylose reached 94% and 68%, respectively (run 7). Moreover, the addition of xylanase shortened the saccharification time of xylan to 168 h, as shown in Figure 1.

### SSCF of the LMAA-pretreated napiergrass

The results of SSCF were listed in Table 2. The ethanol yield was 43% in the SSCF of non-treated napiergrass using *S. cerevisiae*, *E. coli* KO11, cellulase and xylanase, (Table 2, run 1). The LMAA-pretreatment enhanced the ethanol yield (runs 2–3). The amount of napiergrass was optimized to be 3.0 g from the comparison of ethanol yields in the cases of 2.5 to 4.0 g (runs 5–7). Maximum ethanol yield was 74% in SSCF of LMAA-pretreated napiergrass using *S. cerevisiae*, *E. coli* KO11, cellulase, and xylanase for 96 h (run 2). The ethanol yield was not changed even though SSCF time was elongated until 168 h when the xylose yield reached the maximum yield in saccharification process. In the case of SSCF without *S. cerevisiae* (run 3), the ethanol yield was 70%. If the glucan (1.191 g) occurring in the LMAA-pretreated napiergrass (3 g) is completely turned to ethanol, 676 mg of ethanol will be produced. Ethanol amounts of SSCF in runs 2 and 3 exceeded this value (676 mg), showing that the pentose fermentation apparently occurred. Moreover, the ethanol amount (777 mg, run 2) was comparable to the ethanol amount (880 mg) which was ethanol amount when glucose and xylose produced by enzymatic saccharification (Table 1 run 7) would be completely fermented. Therefore, it was suggested that yields of hexose and pentose fermentations were moderately high.

**Table 2:** **SSCF of LMAA-treated napiergrass** ^**a)**^

No	Napiergrass		Enzyme/mg ^d)^	KO11/mL ^e)^	Yeast/mL ^f)^	Products/mg (Yield/%) ^b)^			Ethanol/gL ^-1 h)^
	PT ^c)^	Weight/g				Xylose	Glucose	Ethanol	
1^i)^	NO	3.0	300	21	0.36	209 ± 14 (35)	19 ± 2 (2)	353 ± 31 (43)	8.8
2	LMAA	3.0	300	21	0.36	73 ± 25 (10)	47 ± 10 (4)	777 ± 15 (74)	19.4
3	LMAA	3.0	300	21	0	148 ± 11 (20)	43 ± 2 (3)	731 ± 12 (70)	18.3
4^j)^	LMAA	3.0	300	0	0.36	473 ± 43 (65)	29 ± 10 (2)	576 ± 39 (55)	14.4
5	LMAA	2.5	250	21	0.36	304 ± 15 (50)	37 ± 6 (3)	547 ± 41 (63)	13.7
6	LMAA	3.5	350	21	0.36	385 ± 45 (53)	37 ± 2 (3)	719 ± 13 (59)	18.1
7	LMAA	4.0	400	21	0.36	438 ± 37 (60)	39 ± 8 (3)	829 ± 28 (60)	21.4

^a)^SSCF was performed for napiergrass (3.0–4.0 g) using cellulase (150–200 mg) and xylanase (150–200 mg) in buffer (19 mL), *E. coli* KO11 (21 ml), and *S. cerevisiae* (0.36 mL) at 36°C for 96 h. The data were expressed as averages of the experiments at three times.

^b)^The maximum amounts of xylose, glucose, and ethanol were 729 mg, 1323 mg, and 1049 mg obtained from 3.0 g of LMAA-pretreated napiergrass, respectively.

^c)^Pretreatment (PT). NO: non-treatment. LMAA: a low-moisture anhydrous ammonia pretreatment for four weeks.

^d)^Weight of total hydrolytic enzyme (cellulase and xylanase) in mg. The fraction (*F*_X_) of xylanase in the mixture of cellulase and xylanase was 0.50.

^e)^Volume of the cell suspension of *E. coli* KO11 in ml.

^f)^Volume of the cell suspension of *S. cerevisiae* in mL.

^h)^Concentration of ethanol in g L^-1^.

^i)^The maximum amounts of xylose, glucose, and ethanol obtained from non-treated napiergrass (3.0 g) were 576 mg, 1043 mg, and 827 mg, respectively.

^j)^The simultaneous saccharification and fermentation (SSF) using cellulase (150 mg) and xylanase (150 mg), and *S. cerevisiae* (0.36 mL) at 36°C for 96 h.

### The additive effect of *Saccharomyces cerevisiae*

*S. cerevisiae* is the most commonly used microorganism for industrial ethanol production. However, it cannot utilize xylose for growth and ethanol production. Therefore, it is requisite to use a recombinant species which can ferment pentose. In our SSCF process, *S. cerevisiae* was used in addition to a recombinant *E. coli* KO11 for fermentation, since it was found that the SSCF using four components (*S. cerevisiae*, *E. coli* KO11, cellulase, and xylanase) proceeded slightly faster than the SSCF without S. cerevisiae.

**Figure 3:**
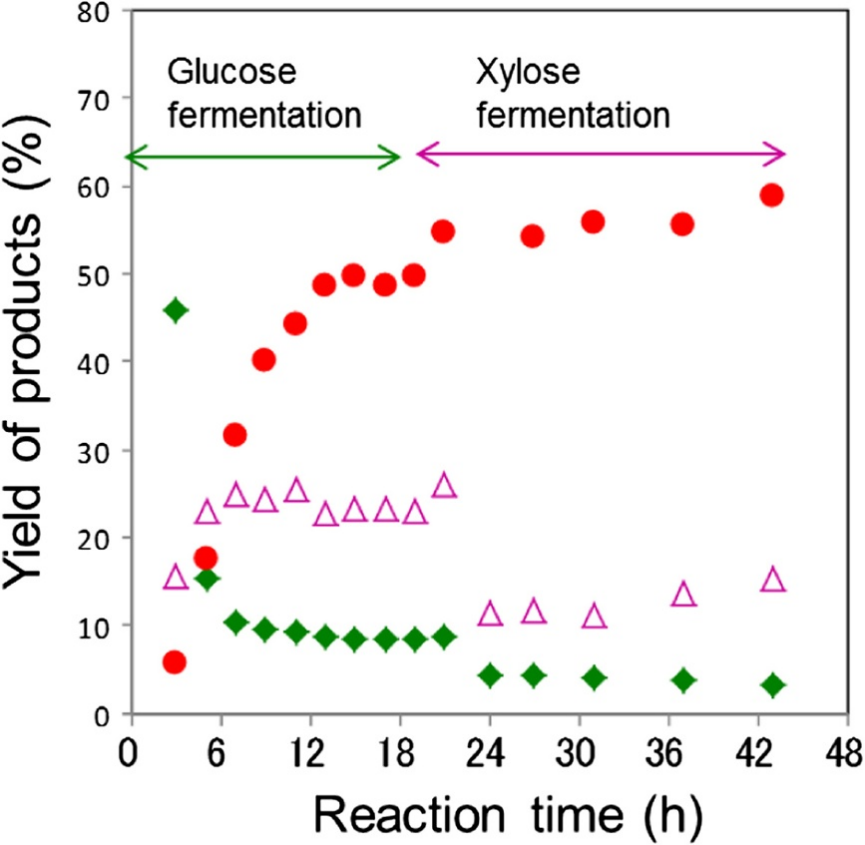
**Time-conversion of yields of ethanol (**●**), glucose (**◆**), and xylose (** **△** **) in the SSCF using** ***S. cerevisiae*** **,** ***E. coli*** **KO11, cellulase, and xylanase (Table** 2 **run 2).**

Figure 3 shows time conversions of ethanol, glucose, and xylose in SSCF. The ethanol yield increased gradually until the yield reached 50% which was comparable to the SSF yield (55%) using *S. cerevisiae*, cellulase and xylanase (Table 2, run 4). At that time, the glucose was completely fermented due to the fast glucose formation in the saccharification while the xylose was accumulated due to slow fermentation of xylose. After SSCF for 18 h, the xylose fermentation started, resulting in that ethanol yield increased again.

## Conclusions

**Figure 4:**
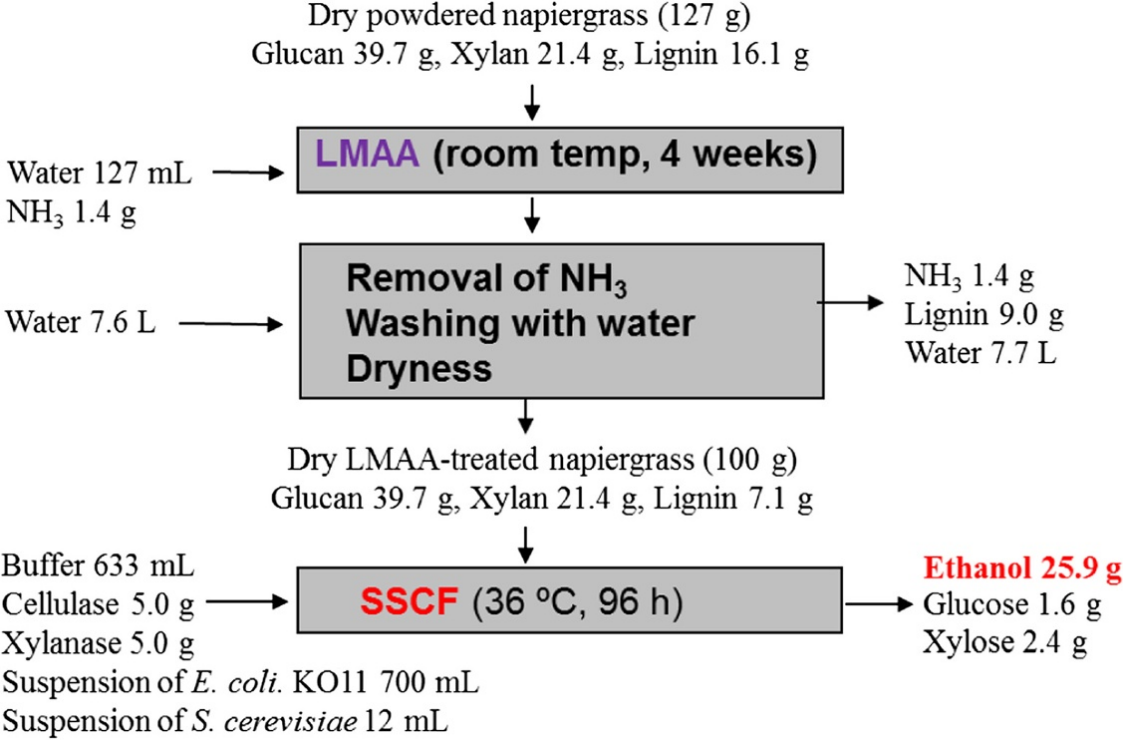
**Mass balance of SSCF process of the LMAA-pretreated napiergrass.**

We have previously examined ethanol formation through a combination of SSF using enzyme and *S. cerevisiae* with a pentose fermentation using *E. coli* KO11. The ethanol yields from LMAA-pretreated and non-treated napirgrass were 69% (Yasuda et al. 2013) and 44% (Yasuda et al. 2012), respectively. The present SSCF process improved the ethanol yield to 74%. Mass balance of the present SSCF is summarized in Figure 4. Consequently 25.9 g of ethanol was produced from 100 g of LMAA-pretreated napiergrass which was obtained from 127 g of the non-treated powdered napiergrass. Since the SSCF can be performed in one-pot reaction, it can prevent contamination risks of other micro-organism and can construct simple processing procedure. Thus, efficient bio-ethanol production from napiergrass was successfully achieved by the combination of the LMAA-pretreatment with the SSCF process using four components (cellulase, xylanase, *Saccharomyces cerevisiae*, and *E. coli* KO11).
